# Microbiota as a biomarker of tuberculosis treatment response: association with clinical history, inflammation and outcome

**DOI:** 10.21203/rs.3.rs-11050242/v1

**Published:** 2026-09-30

**Authors:** Grant Theron, Charissa Marsh, Loide Shipingana, Adam Cantor, Suventha Moodley, Happy Tshivhula, Tariq Webber, Zaida Palmer, Tinaye Chiyaka, Hilary Monaco, Benjamin Wu, Yonghua Li, Destiny Collazo, Shivani Singh, Robin Warren, Leanie Kleynhans, Jose Clemente, Leopoldo Segal

**Affiliations:** Stellenbosch University; Stellenbosch University; Stellenbosch University; Icahn School of Medicine; Stellenbosch University; Stellenbosch University; Stellenbosch University; Stellenbosch University; Stellenbosch University; Mount Sinai School of Medicine; New York University School of Medicine; New York University Grossman School of Medicine; NYU Grossman School of Medicine Division of Pulmonary, Critical Care, and Sleep Medicine; New York University; Stellenbosch University; Mater Research - The University of Queensland; Icahn School of Medicine at Mount Sinai; New York University

**Keywords:** microbiota, tuberculosis, antibiotic treatment, immune response, outcomes

## Abstract

Tuberculosis requires prolonged multidrug therapy. Whether this disrupts the microbiota in ways reflecting previous treatment exposure and outcome is unknown. We performed 16S rRNA gene sequencing on sputum and stool from 107 people with tuberculosis (cases) in a longitudinal cohort, sampled at baseline, month 2, end-of-treatment (month 6), and months 12 and 18, and ≤2 close contacts per case (n=180) at baseline, months 6 and 18. Serum cytokines were quantified in cases (baseline, months 6 and 18) and contacts (baseline). Treatment outcome was defined by month 2 culture-conversion and month 6 culture/smear status. Longitudinal microbial time-series were clustered using LoClust and linked to clinical data. Cases' α-diversity declined initially, recovering by month 6 in sputum and month 12 in stool. Stool β-diversity reductions, driven by Dialister-enrichment and Clostridiales-depletion, persisted to month 18; close contacts remained stable throughout. Unfavourable outcomes showed sputum Pseudomonas- and Mycobacterium gordonae-enrichment and Porphyromonas endodontalis-depletion, and stool Clostridiales-enrichment and Dialister-depletion. Clustering identified five α-diversity trajectories, with declining-diversity trajectories enriched for previous TB. Microbiota-inflammation associations persisted beyond treatment, with Clostridiales independently predicting IP-10 and TNF-α, and Dialister predicting C-reactive protein. Microbiota recovery trajectories are linked to previous tuberculosis, and specific taxa to outcome and inflammation, highlighting biomarker potential.

## Introduction

Over 40 million people were treated for tuberculosis (TB) in the last five years^1^. TB is one of few diseases requiring prolonged antibiotic exposure (≥ 4 antibiotics for at least 6 months)^2^. While short-term treatment-associated depletion of the commensal microbiota is described^3,4^, there are, to our knowledge, few data on long-term perturbations after treatment cessation. We hypothesised that these perturbations persist beyond treatment, that microbiota responses to treatment differ between individuals, and that these microbial trajectories are associated with specific immunological markers.

Antibiotic-mediated microbial disruption is linked to an increased risk of childhood-onset asthma^5^, obesity^6^, and diabetes^7,8^. First-line TB treatment comprises isoniazid (H), rifampicin (R), pyrazinamide (Z), and ethambutol (E) taken daily for the first two months (intensive phase), followed by four months with HR only (continuation phase). Rifampicin is traditionally considered the only broad-spectrum antibiotic in the regimen, primarily targeting *Mycobacteria*^9,10^; however, drugs like HZE, which were considered to have narrow spectrum, have distinct effects on the murine gut microbiota when given individually or in different combinations^9^. In humans, TB treatment reduces microbial diversity as early as two weeks in stool^4,9,11^ and two months in sputum^12^, depleting taxa with essential immunomodulatory functions. For example, *Clostridia* spp., which are depleted by HRZE^4,9,13^, are known gut anaerobic fermenters that increase regulatory T cell induction^14^ via short-chain fatty acid (SCFA) production.

Here, we characterised sputum and stool microbiota changes in people with TB (including those living with HIV; PLHIV) before, during, and after TB treatment. Specifically, we examined whether microbial changes during treatment are associated with favourable or unfavourable outcomes. We also investigated whether cases clustered distinctly based on their microbial trajectories over time, and whether this was influenced by clinical history. Finally, we assessed cytokine responses in serum from cases and close contacts (baseline only) and inferred microbial-immune relationships before, at the end and after treatment.

## Methods

### Ethics

The study was conducted in accordance with the Declaration of Helsinki and approved by the Stellenbosch University Health Research Ethics Committee (N14/10/136). All participants provided written informed consent.

### Study participants

Participants were sourced from a prospective cohort as described (13 April 2016–15 September 2021)^3,15^. Adults (≥ 18 years) who were sputum *Mycobacterium tuberculosis* complex (MTBC) culture-positive and had not been on treatment within the last two months were eligible for microbiota sampling. Participants were sampled at diagnosis (baseline), during treatment (month 2), the end-of-treatment (month 6) and post-treatment (months 12 and 18) (Fig. 1A). Two culture-negative, adult close contacts who had not taken antibiotics in the last three months and preferably from the same household were identified (**Supplementary Methods**). We enrolled an alternative contact if a close contact became TB-positive during the study. Close contacts were seen at baseline, months 6 and 18. We collected demographic and clinical data for all participants.

### Treatment outcomes

At month 2, treatment outcomes were defined by sputum culture conversion, with cases classified as culture-negative or culture-positive for MTBC, excluding contaminated (indeterminate) results.

At month 6, a favourable outcome was a negative sputum culture or, if culture was contaminated or unavailable, two consecutive negative sputum smears. An unfavourable outcome was a sputum culture positive for MTBC or at least one acid-fast bacilli-positive sputum smear if the culture was negative or contaminated.

### Specimen collection and processing

We obtained an induced sputum and stool specimen at each visit as described^3^. Three sputa were collected for microbiota and TB testing. Induced sputum was decontaminated with *N*-acetyl-*L*-cysteine (sodium hydroxide omitted to limit harsh exposure to microbiota) on the day of collection, pelleted (3217×*g*), and resuspended in 2 mL phosphate buffer (pH 6.8; BD, Johannesburg, South Africa) before biobanking (−80°C). Stool was received in a tube containing PSP DNA stabiliser the day after collection and frozen (−20°C). Whole blood was collected in clot activator vacutainers (BD), centrifuged (2000×*g*, 10 min), and serum stored (−80°C) for cytokine measurement.

### DNA extraction and sequencing

Microbial DNA was extracted from sputum using the Purelink Microbiome DNA Purification Kit (Invitrogen, Carlsbad, USA) and stool using the PSP Spin Stool DNA Plus Kit (Stratec Biomedical, Birkenfeld, Germany) as per manufacturer’s instructions^16^. Bacterial 16S rRNA gene sequencing (V4 region, 150 bp, paired-end) was done on the Illumina MiSeq as described^3^.

### Microbiota analysis

Sequences were pre-processed in QIIME2 (v.2.2020) as described^17^ and analysed in R (v.4.3.2; R Foundation for Statistical Computing, Vienna, Austria). α-Diversity (Shannon index) and β-diversity^18^ (Bray-Curtis dissimilarity index) were calculated using vegan^19^ (v.2.6–4). Differential abundance testing was done at amplicon sequence variant (ASV) level using edgeR^20^ (v.4.0.12) (**Supplementary Methods**). Non-parametric tests, including the Friedman test and Mann-Whitney U test, were used for α-diversity comparisons. β-Diversity was assessed using permutational multivariate analysis of variance (PERMANOVA). Generalised additive models (GAM) were performed using mgcv^21^ (v.1.9–3) to assess whether α-diversity trajectories differed between cases and close contacts over time, while accounting for unbalanced sampling due to missed visits. Longitudinal clustering of time-series data (Chao1 index) were performed using LoClust^22^ among participants with ≥ 3 timepoints. Clusters were examined for associations with clinical data using χ^2^ test of independence. p ≤ 0.05 was considered significant.

### Luminex

Twenty-three cytokine and other soluble markers^23,24^ (IFN-γ, IP-10, TNF-α, IL-1β, IL-6, IL-17, IL-23, IL-4, IL-10, FGFb, VEGF, CRP, procalcitonin, MMP-1, MMP-2, MMP-8, MMP-9, MMP-12, MMP-13, TIMP-1, TIMP-2, TIMP-3, TIMP-4) were measured using Luminex (R&D Systems, Minneapolis, USA; Millipore, Billerica, USA) according to the manufacturer’s instructions^25,26^ on a Bio-Plex 200 platform (Bio-Rad Laboratories, Hercules, USA). Measurements were performed on sera from cases at baseline, month 6 and month 18 and close contacts at baseline. Samples were randomised across plates to minimise batch effects. Concentrations were determined by five-parameter logistic regression (Bio-Plex Manager v6.1). Values outside the standard curve within the assay detection limits were extrapolated, while out-of-range values were imputed as 10% above the highest or below the lowest measured concentration for each marker^27,28^.

### Cytokine analysis

For cytokine analyses, PERMANOVA and Bonferroni correction for multiple comparisons within groups was performed and visualised on principal component analysis using *vegan* (v.2.6–10). Differences between groups were assessed using the paired Wilcoxon signed-rank test. GAM was used to assess cytokine trajectories over time.

### Microbiota and cytokine co-analysis over time

Redundancy analysis (RDA) was used to assess associations between the microbiota and cytokines in cases. ASV-level taxa were clr-transformed using *microViz*^29^ (v.0.12.1) with the model constrained by differentially expressed cytokines in cases. For each timepoint (baseline, month 6, and month 18), the top 25 taxa identified by edgeR were visualised in the RDA biplot. Associations between ASV-level taxa and cytokine concentrations were further assessed using Spearman’s correlation (p < 0.05).

### Data availability

The data generated in this study have been deposited in the Sequence Read Archive under accession code PRJNA1529187. All other supporting data are available within the articles and its Supplementary Information.

### Code availability

The script used for analysis is available at https://doi.org/10.5281/zenodo.22754539.

## Results

### Cohort

We enrolled 107 cases and 180 close contacts (**Supplementary Fig. 1**). Compared to close contacts, cases were more likely to be male, younger, have HIV, previous TB and greater morbidity (Table 1). At the end of treatment, 92/107 (86%) cases had favourable outcome.

### Pre-treatment microbiota differences between cases and close contacts

We first compared cases’ microbiota to their close contacts. Sputum α-diversity was similar (**Supplementary Fig. 2**), but β-diversity differed with cases’ sputum *Porphyromonas endodontalis-*enriched in and *Enterobacteriaceae*-depleted of (Fig. 1). In stool, both α-diversity and β-diversity differed, with cases’ *Neisseria cinerea*-enriched and *Dialister*-depleted. β-diversity differences persisted after adjusting for covariates (**Supplementary Table 1**).

### The sputum microbiota is temporarily affected by TB treatment

#### α-Diversity:

We next evaluated the effects of treatment (Fig. 2A). During the first two months, α-diversity declined in cases (p < 0.001 versus. baseline) and recovered from month 6 onward (p > 0.999). α-Diversity was stable in close contacts (they were not placed on treatment; p = 0.795).

#### β-Diversity and differential taxa over time:

Shifts were apparent as early as month 2 in cases (Fig. 2B–F). No differences were observed between month 2, when the regimen changes, and month 6. Relative to baseline, β-diversity changes persisted at month 6 in cases but recovered by month 12, with no further changes at month 18. At baseline, cases were enriched with *Neisseria cinerea* relative to months 2 and 6 and post-treatment (months 12 and 18; Fig. 2G). Compared to baseline, post-treatment sputum was *Fusobacteriaceae*-enriched.

#### β-Diversity and differential taxa across groups:

At month 6, β-diversity differed between cases and close contacts (p = 0.001) with cases *Prevotella tannerae*-enriched and *Streptococcus*-depleted (**Supplementary Fig. 3**). Differences in β-diversity were sustained at month 18 (p = 0.011) with cases *Moraxella ovis*-enriched and *Biberstenia*-depleted versus close contacts.

TB treatment causes long-lasting microbial perturbations in stool with Clostridiales depletion

#### α-Diversity:

Trajectories varied between cases and close contacts (Fig. 3A) with α-diversity declining by month 2 in cases (p < 0.001) and remaining low at month 6 (p = 0.001) before recovering post-treatment (month 12: p = 0.154, month 18: p = 0.569). α-Diversity was stable in close contacts (p = 0.482).

#### β-Diversity and differential taxa over time:

Shifts were observed from month 2 until month 18 in cases (Fig. 3). No shifts occurred in close contacts’ stool (**Supplementary Fig. 4**). Compared to baseline, cases’ stool showed persistent *Dialister*-enrichment and depletion of seven *Clostridiales* ASVs: five during treatment (months 2 and 6), one throughout (months 2–18), and one post-treatment (months 12–18); close contacts showed no consistent changes (Fig. 3G, **Supplementary Fig. 5**).

#### β-Diversity and differential taxa across groups at specific timepoints:

At month 6, β-diversity differed between cases and close contacts (p = 0.003), with cases *Bacteroidales*-enriched and *Clostridiales-*depleted (**Supplementary Fig. 6**). β-diversity differences were sustained at month 18 (p = 0.021), with cases *Veillonella parvula-*enriched and *Treponema*-depleted versus close contacts.

### Distinct sputum and stool microbiota are associated with treatment outcomes

#### Treatment outcome classifications

61/97 (63%) were culture-negative, 21 (22%) were culture-positive and 15 (15%) were unclassifiable at month 2. At month 6, 92/107 (86%) had a favourable outcome, 11 (10%) an unfavourable outcome and 4 (4%) were unclassifiable.

At month 2, there were no differences in sputum α- (p = 0.387) or β-diversity (p = 0.223) between culturenegatives and culture-positives, nor were there differential taxa. However, stool α-diversity was higher in culture-positives (p = 0.039), and although no β-diversity differences occurred, stool was *Fusobacteriaceae*-enriched and depleted of seven *Prevotella* ASVs compared to culture-negatives (Fig. 4A).

At month 6, only β-diversity differed by outcome. Unfavourable outcomes were associated with *Pseudomonas* and *Mycobacterium gordonae* enrichment and *Porphyromonas endodontalis*-depletion in sputum (Fig. 4B), while stool was *Clostridiales*-enriched and depleted of several *Dialister* spp. (Fig. 4C).

### Cluster analysis reveals distinct α-diversity trends in cases by previous TB status

#### Sputum:

Cases were grouped into five α-diversity trajectory clusters over time (*n* = 91): Clusters 1 (*n* = 16), 2 (*n* = 21), 3 (*n* = 18), 4 (*n* = 23), and 5 (*n* = 13) (Fig. 5A). The clusters had distinct average trajectories. In all clusters, α-diversity declined by month 2 (consistent with earlier analysis; Fig. 2). However, two clusters (Clusters 1 and 4) had a subsequent increase in α-diversity, two plateaued (Clusters 2 and 3), and one (Cluster 5) decreased. Cluster membership was associated with previous TB status (χ^2^=12.44, p = 0.014). Cluster 5 had the highest proportion of previous TB cases (10/13, 77%) and the lowest in Cluster 4 (5/23, 22%).

#### Stool:

Five clusters were identified (*n* = 87): Cluster 1 (*n* = 23), Cluster 2 (*n* = 19), Cluster 3 (*n* = 22), Cluster 4 (*n* = 10), and Cluster 5 (*n* = 13) (Fig. 5B). Cluster membership was again associated with previous TB status (χ^2^=10.44, p = 0.034), with Cluster 4 having the highest proportion of previous TB cases (8/10, 80%) and the lowest in Cluster 1 (6/23, 26%).

### Cytokines elevated in cases at baseline decline with treatment

#### Cytokine expression across groups

At baseline, cytokine concentration differed between cases and close contacts (R^2^ = 0.088, p = 0.001). Cases had higher IP-10, TNF-α, IL-1β, IL-6, IL-23, IL-4, FGFb, VEGF, CRP, procalcitonin, MMP-1, MMP-8, MMP-9, MMP-12, MMP-13 and TIMP-1, and lower MMP-2 than close contacts (**Supplementary Fig. 7A-B**). By month 18, IL-4 and procalcitonin, elevated in cases at baseline, were now lower than in close contacts (p < 0.001 and p = 0.003, respectively), alongside IL-10 and TIMP-3 (p = 0.006 and p < 0.001, respectively; **Supplementary Fig. 8**).

#### Cytokine expression within groups

In cases, overall cytokine concentration changed from baseline to month 6 and from baseline to month 18 (both p = 0.003). Most cytokines declined with treatment (IP-10, TNF-α, IL-1β, IL-6, IL-23, IL-4, IL-10, FGFb, VEGF, CRP, procalcitonin, MMP-1, MMP-8, MMP-9, MMP-12, MMP-13, TIMP-1 and TIMP-3); whereas MMP-2 increased (**Supplementary Fig. 7C-D**).

### Microbiota-cytokine associations resolve with treatment in sputum but persist post-treatment in stool

#### Sputum

At baseline, *Neisseria cinerea* was associated with IL-23, IL-6, IL-4 and procalcitonin, while *Prevotella* and *Veillonella dispar* were associated with CRP, and *Streptococcus* with TNF-α and IP-10 (Fig. 6A–C). By month 6, *Veillonella* was associated with TNF-α, IP-10, IL-23 and procalcitonin. At month 18, *Streptococcus* was associated with procalcitonin. Of these, *Prevotella*-CRP (p = 0.035) and *Veillonella dispar*-CRP (p = 0.011) correlations were significant (**Supplementary Fig. 9**).

#### Stool

At baseline, *Clostridiales* was associated with IL-4 and *Ruminococcaceae* (a *Clostridiales* member) with IL-23, procalcitonin and IP-10 (Fig. 6D–F). At month 6, *Acidaminococcus* and *Pyramidobacter piscolens* were associated with IL-6, *Clostridiales*, *Bacteroidetes* and *Bacteroidales* with IP-10, and *Dialister* with CRP; the latter association with CRP persisted at month 18. *Ruminococcaceae* was associated with IP-10 at month 18 and two *Clostridiales* ASVs with TNF-α. Of these, the *Clostridiales*-TNF-α correlation approached significance (p = 0.057).

## Discussion

We show that: 1) before treatment, cases had distinct sputum and stool microbiota compared to close contacts, with depletion of commensal taxa; 2) TB treatment caused α-diversity loss in sputum and stool, especially in the first two months; 3) β-diversity shifts were transient in sputum but lasted at least one year post-treatment in stool, with *Dialister*-enrichment and *Clostridiales* depletion, whereas close contacts’ microbiota remained stable; 4) unfavourable outcomes were associated with higher α-diversity at month 2 and, at month 6, different stool β-diversity and distinct sputum (*Pseudomonas* and *Mycobacterium gordonae* enrichment, *Porphyromonas endodontalis* depletion) and stool microbiota (*Clostridiales* enrichment, *Dialister* depletion); 5) cluster analysis identified five α-diversity trajectories in sputum and stool, with membership associated with previous TB status; 6) treatment resolved most microbiota-cytokine associations in sputum, whereas associations in stool persisted or re-emerged post-treatment, notably *Clostridiales* (including *Ruminococcaceae*) with IP-10 and TNF-α and *Dialister* with CRP.

Our findings support previous observations that the microbiota of cases and close contacts differ before TB antibiotics, with depletion of key respiratory and gut commensals in cases^3,30–32^. Many of these taxa regulate host immunity by producing vitamins^33^ and SCFAs^34^ and their depletion may relate to increased TB disease susceptibility.

Treatment’s effects were most pronounced in the first two months, aligning with cohorts in Africa^12^ and Haiti^4^, likely reflecting the stronger selective pressure of the four-drug intensive phase regimen. Sputum effects were transient, with β-diversity returning to baseline by month 12; however, *Neisseria cinerea*, an upper respiratory commensal^35^, remained depleted throughout, and further studies are needed to assess the implications. Stool shifts were long-lasting, with *Clostridiales* depletion up to one year post-treatment, supporting previous findings^4,36,37^ and likely driven by rifampicin and pyrazinamide^9^. Commensal *Clostridiales* are crucial for immune function, gut barrier integrity, and metabolite production (e.g., SCFAs, bile acids, indolepropionic acid), all relevant for TB. For instance, indole-3-propionic acid from *Clostridium sporogenes* shows antitubercular activity *in vitro*^38^.

Individuals with unfavourable outcomes had higher stool α-diversity at month 2. It is plausible that the absence of early culture conversion (reflecting a slower bactericidal response) also indicates a lower magnitude of gut microbiota disruption. Sputum findings at month 6 are consistent with an earlier study reporting a higher *Pseudomonas*/*Mycobacterium* ratio in treatment failure versus cured groups. *Dialister* spp. are Gram-negative anaerobes whose effects on host immunity could be negative (inflammatory^39^) or positive (via SCFAs^40^), depending on interactions with co-occurring taxa, and have also been identified as prognostic markers of unfavourable outcomes in other diseases^39,41^. Larger, adequately powered studies are needed to validate these taxa as prognostic markers.

A key finding was that cluster membership was associated with previous TB status in sputum and stool, with declining trajectories showing higher proportions of previous TB cases. This suggests previous treatment may predispose the microbiota to incomplete recovery upon subsequent antibiotic exposure^42^, leaving individuals with previous TB more “fragile” to treatment. This is the first evidence linking microbiota recovery to previous TB status, and it may help explain why retreatment cases have poorer outcomes, though this requires further study.

Elevated cytokine concentration in cases versus close contacts at baseline corroborates previous reports for IP-10, IL-6, IL-23, IL-4, CRP and procalcitonin, MMP-1, MMP-8, and MMP-12^43–48^; these are considered markers of bacterial burden and disease severity^49^. The convergence of cytokine concentration following treatment suggests resolution of inflammation^50^, with CRP, IL-4, IL-6 and IP-10 typically declining within a month and MMP-1/MMP-8 by six months, consistent with our findings^51–54^. Despite overall immune recovery, MMP-2 increased over time and may reflect residual inflammation^55^.

Persistent associations between *Ruminococcaceae*, *Clostridiales* and *Dialister* and inflammatory markers until month 18 suggest these stool taxa are linked to inflammation beyond treatment, though whether this reflects a causality, a response to an inflamed gut environment, or both, cannot be determined here.

Strengths of this study include paired sputum and stool sampling in the same individuals; integration of microbiota and cytokine data; and one of the largest longitudinal cohorts to date analysing microbiota shifts during TB treatment. Including close contacts allowed us to account for shared environmental factors (e.g., diet) and helped confirm that microbiota changes in cases were attributable to treatment itself rather than shared exposures. Limitations including: the parent study was a cross-sectional diagnostic study and not powered to assess outcomes, though our findings provide a basis for future, adequately powered investigations; earlier microbiota changes may have been missed, as our data begin after the intensive phase; and functional profiling of stool was not performed, which could have clarified the mechanisms underlying the microbiota-immune relationships identified.

In summary, TB treatment has a lasting impact on the sputum and stool microbiota. We show for the first time that longitudinal microbiota trajectories are linked to previous TB status, and that distinct sputum and stool microbial profiles distinguish individuals with unfavourable treatment outcomes. The persistence of specific microbiota-cytokine associations in stool post-treatment, particularly involving *Clostridiales* and *Dialister* suggests these taxa could hold potential as biomarkers of recovery and treatment response. Confirming this in larger studies, alongside functional profiling and testing across emerging shorter TB regimens^56,57^ that remain reliant on broad-spectrum antibiotics, is necessary.

## Supplementary Files

This is a list of supplementary files associated with this preprint. Click to download.

- STROBEchecklistv4.pdf
- supplement.docx

## Figures and Tables

**Figure 1:**
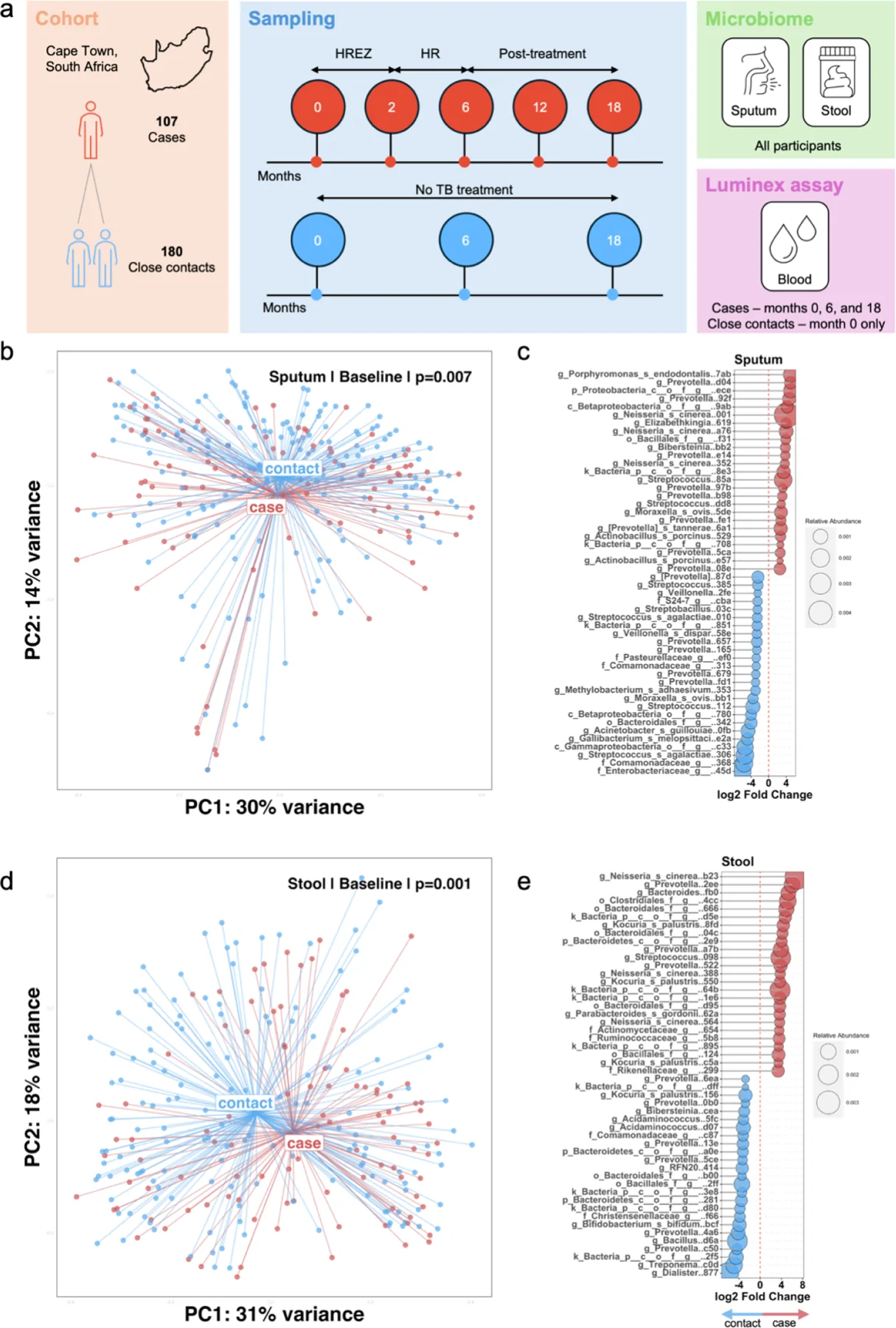
Cases and close contacts have distinct sputum and stool microbiota before treatment. **(A)** Overview of specimen collection and procedures in cases and paired close contacts. **(B)** Distinct sputum β-diversity between groups, **(C)** with cases *Neisseria cinerea*-enriched and *Enterobacteriaceae*-depleted compared to close contacts. Discriminatory taxa appear closer to the threshold (red dotted line, false discovery rate of 0.2); relative abundance correspond to circle size. **(D)** β-diversity also differed in stool, **(E)**with cases *Neisseria cinerea*-enriched and *Dialister*-depleted compared to close contacts. HRZE: isoniazid, rifampicin, ethambutol, pyrazinamide.

**Figure 2:**
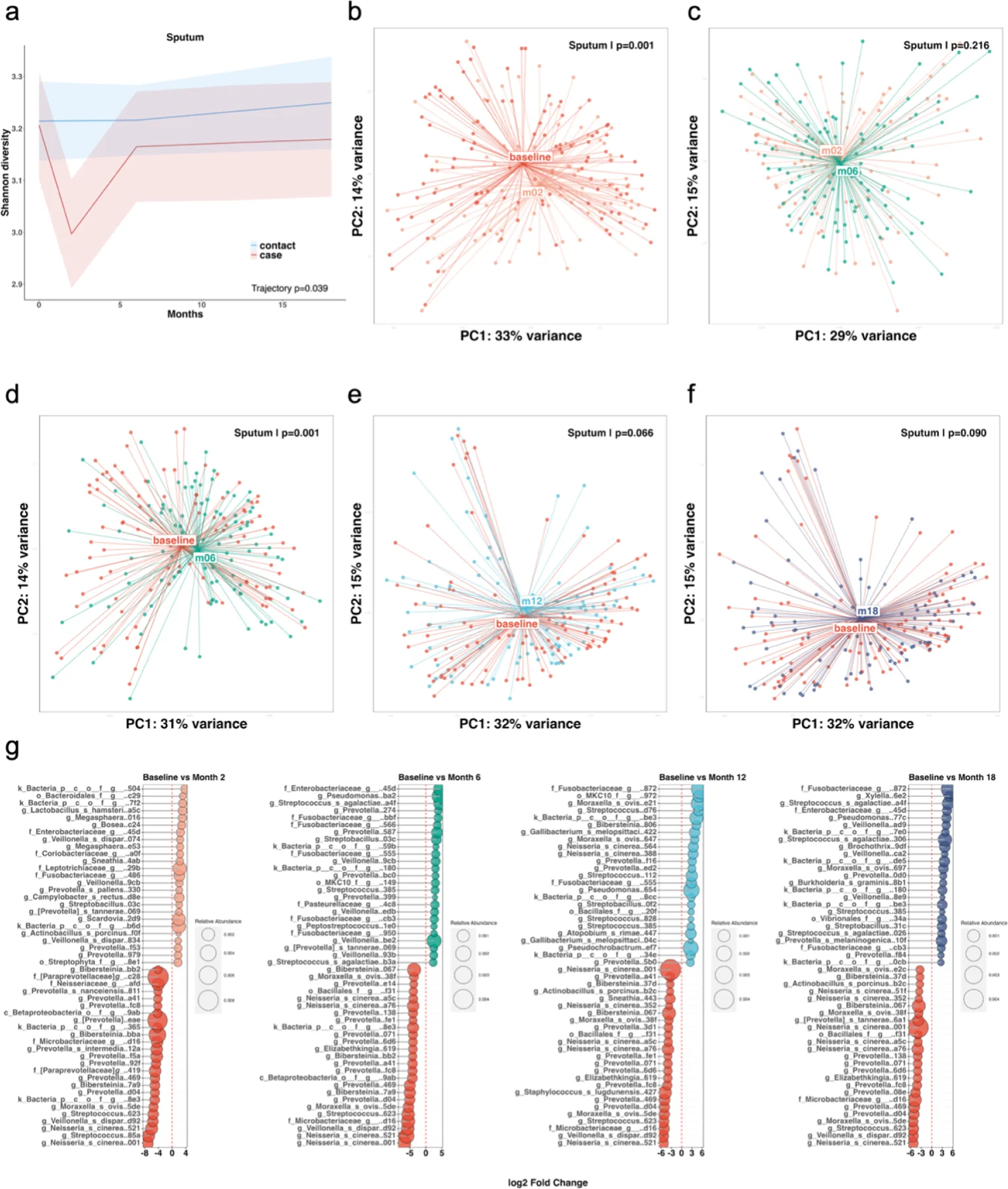
Longitudinal sputum microbiota changes in cases and close contacts. **(A)** Cases and close contacts have distinct α-diversity trajectories: α-diversity declines by month 2 in cases but remains constant in close contacts. **(B-F)** β-Diversity shifts occur only during treatment (months 2 and 6), returning to baseline levels thereafter. Findings were similar after excluding participants with recent non-TB antibiotic exposure (**Supplementary Table 2**). **(G)** Baseline sputa are consistently *Neisseria cinerea-*, *Veillonella dispar-*and *Bibersteinia*-enriched versus follow-up timepoints. Discriminatory taxa appear closer to the threshold (red dotted line, false discovery rate of 0.2); relative abundance correspond to circle size. Red circles represent baseline; orange, green, blue, and navy represent months 2, 6, 12, and 18, respectively.

**Figure 3:**
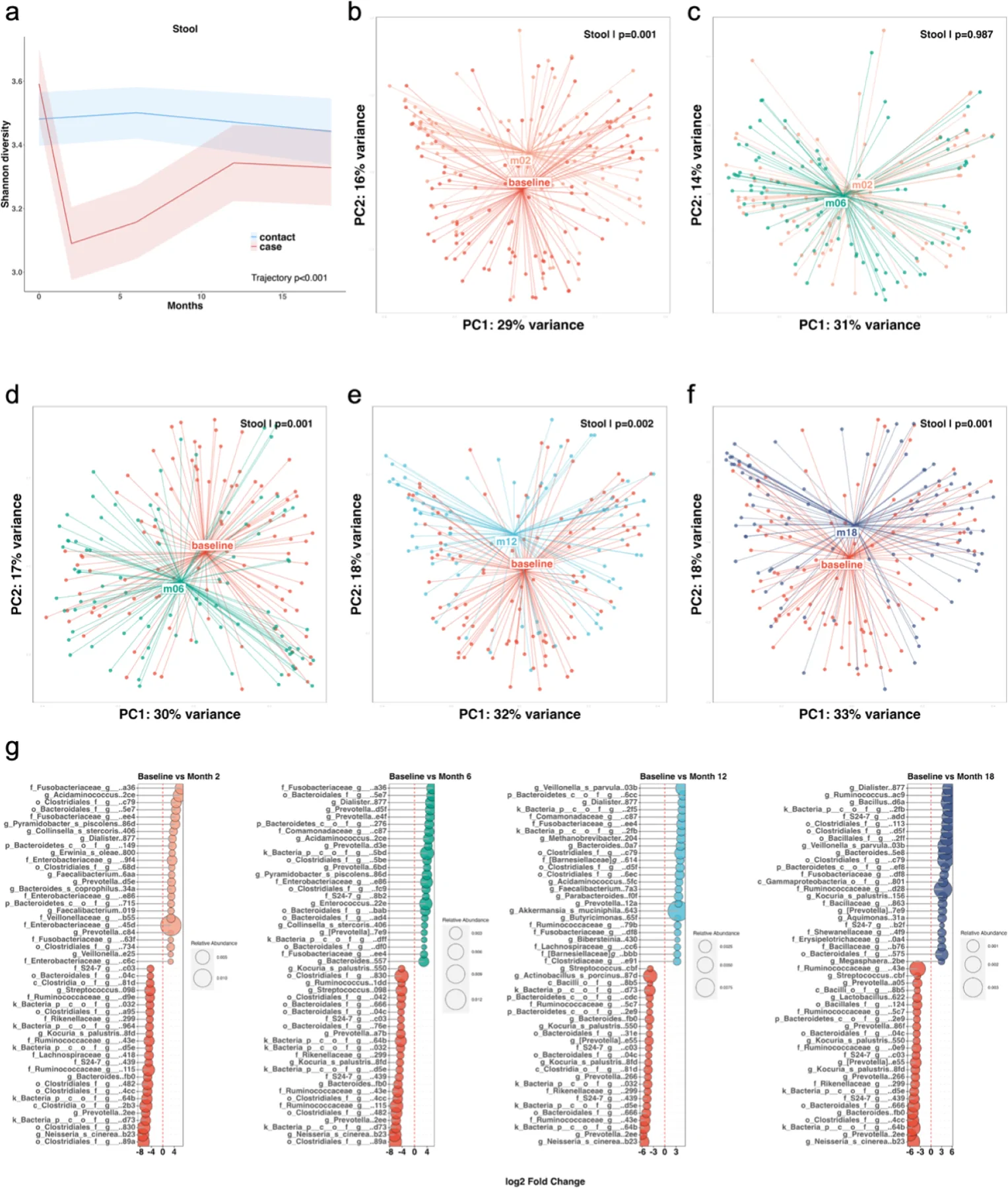
Stool microbiota changes persist until one-year post-treatment in cases. **(A)** Cases and close contacts have distinct α-diversity trajectories: α-diversity declines by month 2 and recovers post-treatment in cases but remains contacts in close contacts. **(B-F)**β-Diversity shifts persist from month 2 through month 18. **(G)** *Clostridiales*is consistently depleted in baseline stool relative to follow-up timepoints. Discriminatory taxa appear closer to the threshold (red dotted line, false discovery rate of 0.2); relative abundance correspond to circle size. Red circles represent baseline; orange, green, blue, and navy represent months 2, 6, 12, and 18, respectively.

**Figure 4:**
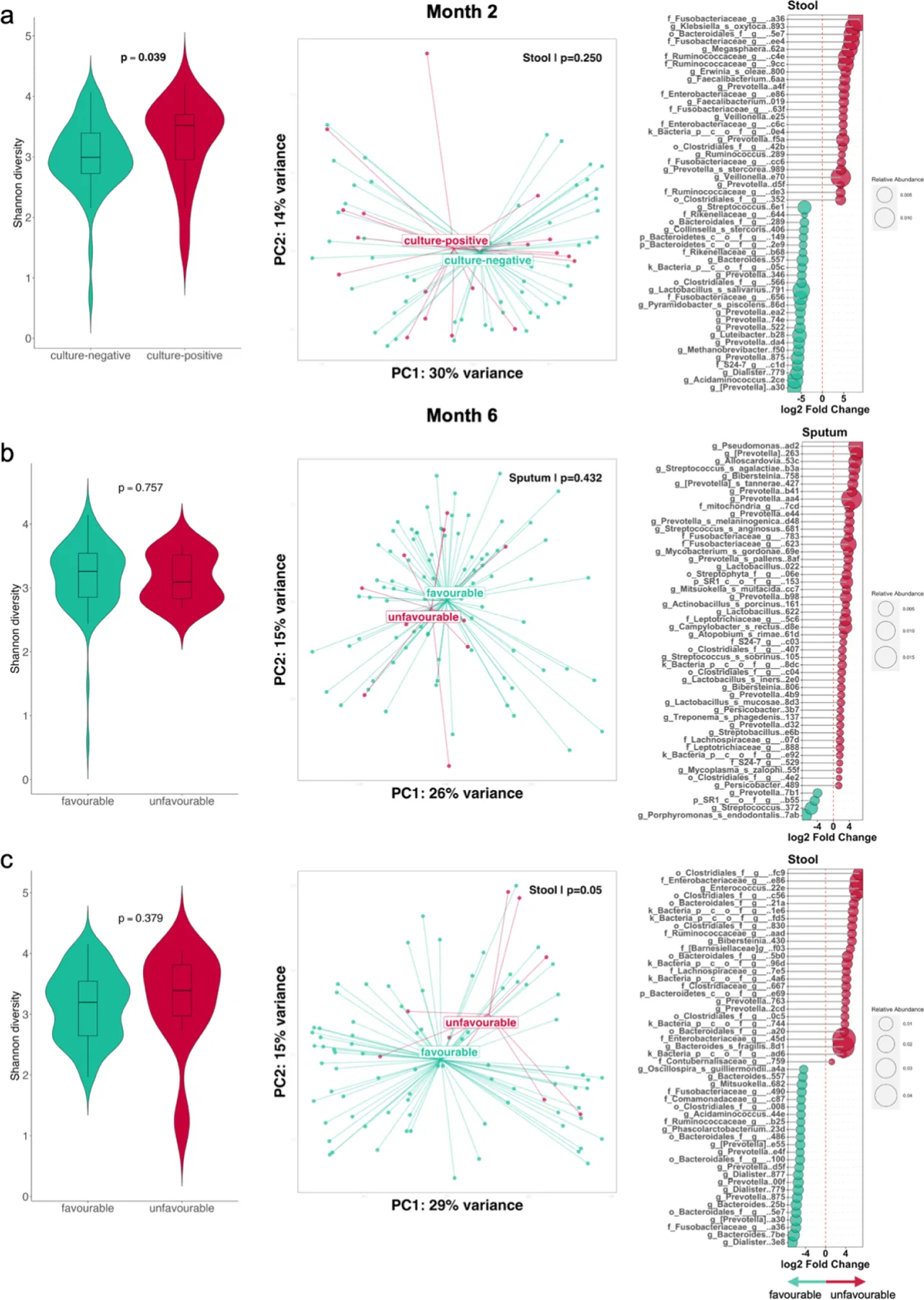
Diversity and taxa differ by treatment outcomes in sputum and stool at months 2 and 6. **(A)** At month 2, culture-positives have higher α-diversity, similar β-diversity and differential taxa compared to culture-negatives. **(B)** At month 6, sputum α- and β-diversity are similar by outcome, but unfavourable outcomes are *Pseudomonas-* and *Mycobacterium gordonae*-enriched and *Porphyromonas endodontalis*-depleted versus favourable outcomes. In stool, α-diversity is similar, but β-diversity differs, with unfavourable outcomes *Clostridiales-*enriched and *Dialister*-depleted.

**Figure 5:**
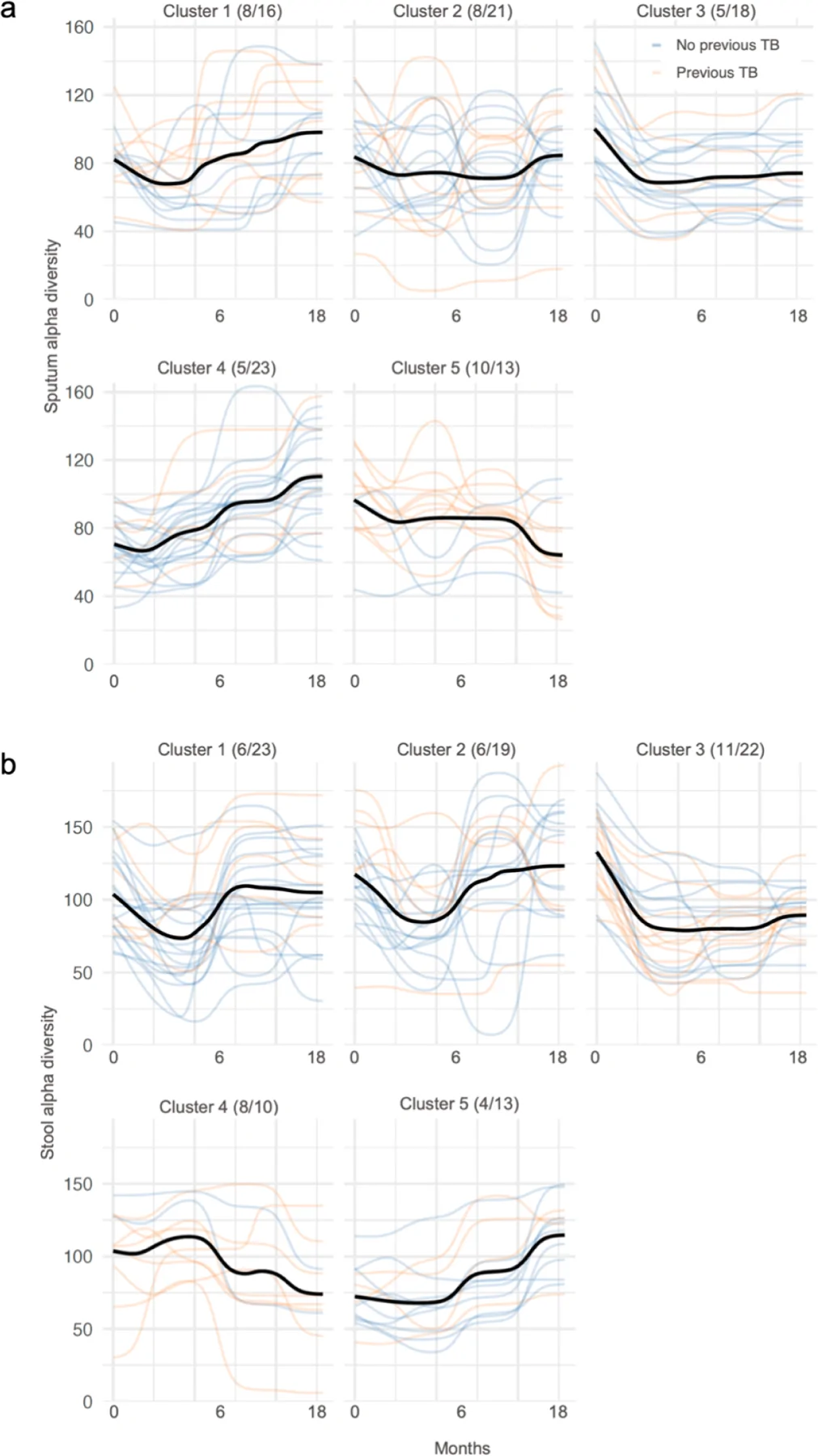
Distinct α-diversity trajectories associated with previous TB status in sputum and stool. Coloured lines represent each case’s α-diversity trajectory over time; black lines indicate the average trajectory per cluster.

**Figure 6:**
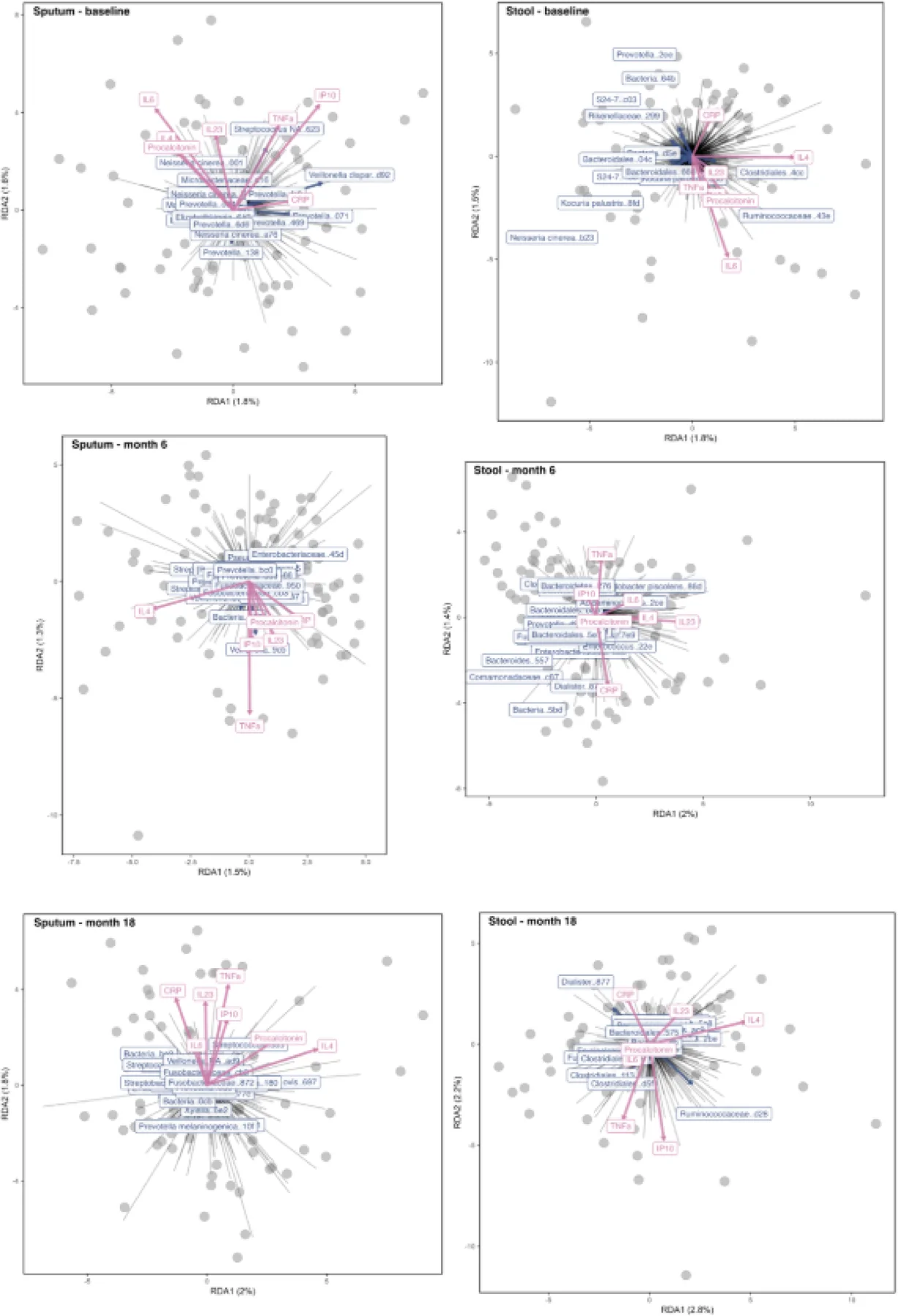
RDA biplot showing associations between sputum and stool microbiota and cytokines at baseline, month 6 and month 18. Grey points represent individual samples; blue arrows represent taxa (enriched per edgeR analyses); pink arrows represent cytokines.

**Table 1:** Study profile. Data are summarised as n (%) or median (IQR).

Characteristics	Cases (n = 107)	Contacts (n = 180)	*p*-value
*Demographics*			
**Males**	52 (49)	60 (33)	**0.015**
**Age (years)**	32 (24–43)	38 (28–53)	**0.001**
**Ethnicity**			
Black	11 (10)	10 (6)	0.211
Mixed ancestry	96 (90)	170 (94)	
**Clinical**			
**HIV**			**0.001**
Negative	80 (75)	150 (90)	
Positive	27 (25)	16 (10)	
**ART**			0.595
No	13 (54)	6 (40)	
Yes	11 (46)	9 (60)	
**Previous TB**			**< 0.001**
No	57 (59)	153 (85)	
Yes	40 (41)	27 (14)	
Months since prior treatment completion	54 (36–97)	83 (43–192)	
**TBScore II**			**< 0.001**
Mild (0–5)	90 (84)	154 (100)	
Severe (≥ 6)	17 (16)	0	
**Non-TB antibiotics**			**0.023**
No	48 (81)	166 (93)	
Yes	11 (19)	13 (7)	
**Self-reported substance abuse**			
**Fagerström dependence score**			**0.018**
Low to moderate (0–4)	35 (33)	86 (48)	
Moderate to high (≥ 5)	72 (67)	94 (52)	
**AUDIT score**			0.691
Low to excessive (0–15)	92 (86)	159 (88)	
Harmful to dependant (≥ 16)	15 (14)	21 (12)	
**Narcotic usage**	32 (30)	35 (20)	0.063
**Adverse events during treatment**		-	
No	81 (84)		
Yes	16 (16)		
Itching	10 (63)		
Poor appetite, nausea or vomiting	5 (31)		
Skin rash	3 (19)		
Loose stool	1 (6)		
Insomnia	1 (6)		

Missing data: HIV (*n* = 14); ART (*n* = 4); previous TB (*n* = 10); TBScore II (*n* = 26); antibiotics (*n* = 49); narcotics (*n* = 1).
